# O-GlcNAcylation of PFKFB3 is required for tumor cell proliferation under hypoxia

**DOI:** 10.1038/s41389-020-0208-1

**Published:** 2020-02-14

**Authors:** Yinrui Lei, Tao Chen, Yeyi Li, Man Shang, Yan Zhang, Yuepeng Jin, Qiujing Yu, Fang Guo, Ting Wang

**Affiliations:** 1 Department of Pharmacology And Tianjin Key Laboratory of Inflammation Biology, School of Basic Medical Sciences, Tianjin Medical University, Tianjin, 300070 China; 2 Key Laboratory of Systems Biomedicine (Ministry of Education), Shanghai Center for Systems Biomedicine, Shanghai Jiao Tong University, 800 Dongchuan Road, Shanghai, 200240 China; 3 Endoscopy Center, Shanghai East Hospital, Tongji University, 150Jimo Road, Shanghai, 200120 China; 4 Department of General Surgery, the First Affiliated Hospital of Wenzhou Medical University, Wenzhou, 325000 China; 5 Key Laboratory of Immune Microenvironment and Disease (Ministry of Education), Department of Immunology, School of Basic Medical Sciences, Tianjin Medical University, Tianjin, 300070 China; 6 The Institute of Cell Metabolism and Disease, Shanghai Key Laboratory of Pancreatic Cancer, Shanghai General Hospital, School of Medicine, Shanghai Jiaotong University, Shanghai, 201620 China

**Keywords:** Glycosylation, Cancer metabolism

## Abstract

The protein O-GlcNAcylation catalysed by O-GlcNAc transferase (OGT) is tightly regulated by glucose availability. It is upregulated and essential for tumor cell proliferation under hypoxic conditions. However, the mechanism behind is still unclear. Here, we showed that the glycolytic regulator 6-phosphofructo-2-kinase/fructose-2,6-bisphosphatase (PFKFB3), which also promotes cell cycle progression in the nucleus, was O-GlcNAcylated in response to hypoxia. The O-GlcNAcylation of PFKFB3 could compete phosphorylation by hypoxia-activated ERK at the same modification site Ser172. Phosphorylated PFKFB3 could interact with the protein G3BP2 and retain in the cytosol; this in turn led to the accumulation of hypoxia-induced-P27 in the nucleus resulting in the cell cycle arrest. Such a pathway was compromised by high level of PFKFB3 O-GlcNAcylation in tumor cells contributing to cell cycle progression. Consistently, the PFKFB3-Ser172 phosphorylation level inversely correlated with the OGT level in pancreatic cancer patients. Our findings uncovered an O-GlcNAcylation mediated mechanism to promote tumor cell proliferation under metabolic stress, linking the aberrant OGT activity to tumorigenesis in pancreatic cancer.

## Introduction

Cancer cells need to reprogram signaling pathways for cell proliferation to resist microenvironment stress with limited oxygen and glucose, presumably through the altered post-translational modification of functional proteins^1^. Cellular O-GlcNAcylation, which is reversibly catalyzed at protein Ser/Thr residues by O-GlcNAc transferase (OGT) and O-GlcNAcase (OGA)^2^, is tightly regulated by the availability of oxygen and glucose^3,4^. Moreover, elevated O-GlcNAcylation levels have been generally reported to be essential for various kinds of tumor development^5–7^. However, it’s still unclear whether and how aberrant O-GlcNAcylation endues cancer cells with the potential to undermine the adverse signals induced by metabolic stress.

Metabolism is fundamentally linked to various cellular physiological events^8,9^. Growing evidence demonstrates that altered metabolic enzymes or metabolites can modulate cellular activities during stress, via directly mediating signaling pathways^10–13^. 6-phosphofructo-2-kinase/fructose-2,6-bisphosphatases 3 (PFKFB3), the hypoxia-induced glycolytic activator, resides in both cytosol and nucleus, and phosphorylates fructose 6-phosphate (F6P) to fructose-2,6-bisphosphate (F2,6BP)^14,15^. The cytosolic PFKFB3 activates the key glycolytic enzyme 6-phosphofructo-1-kinase (PFK1) and guarantees the cellular energy production^16,17^. However, the nuclear PFKFB3 was reported to maintain cell cycle progression via degrading cell cycle inhibitor P27, without affecting the glucose catabolism^18,19^, which obviously accelerates the cellular energy consumption. However, how the multifaceted effects of PFKFB3 are coordinated remains elusive.

In the present study, we found not only the expression level but also the O-GlcNAcylation of PFKFB3 could be induced by hypoxia. However, with limited OGT activity, hypoxia-activated ERK could phosphorylate PFKFB3 at the identified O-GlcNAcylation site, which promotes PFKFB3-G3BP2 interaction and results in PFKFB3 cytosolic retention. Moreover, the O-GlcNAcylation of PFKFB3 with a remarkable level in cancer cells compromises the hypoixa-induced ERK-PFKFB3-G3BP2 pathway and impedes hypoxia-induced P27 accumulation, resulting in cell cycle progression under hypoxia stress condition.

## Results

### PFKFB3 is dynamically modified by O-GlcNAc

**Fig. 1:**
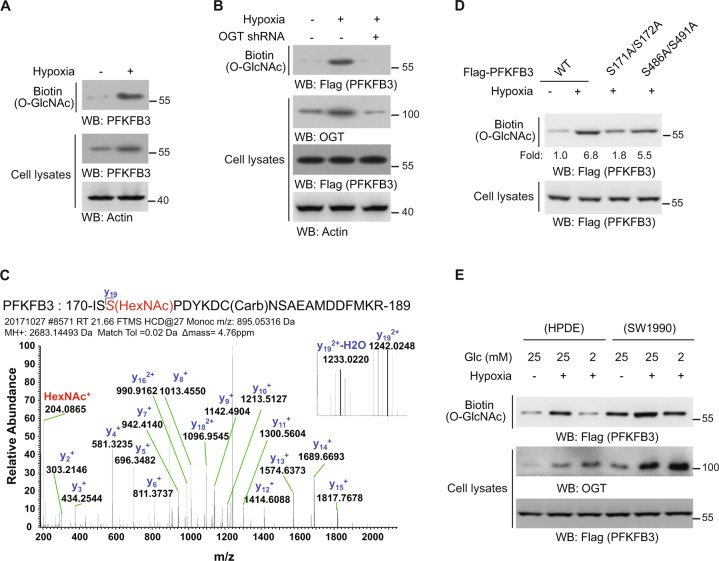
PFKFB3 is modified by *O*-GlcNAc. **a**, **b** SW1990 cells (**a**) with Flag-PFKFB3 and OGT shRNA expression (**b**) were cultured for 12 h under hypoxia or normoxia. The O-GlcNAc modified proteins modified by azide were labeled with biotin and isolated with streptavidin beads for immunoblotting analyses. **c** Flag-PFKFB3 was expressed in SW1990 cells. Immunoprecipitation analysis was performed using the anti-Flag antibody, and the extracts were analyzed by mass spectrometry. Precursor mass shift with HexNAc modification, measured with high mass tolerance (5 ppm); existence of signature HexNAc+1 fragment ions in MSMS spectra; existence of site localization ions (y19+) that covers the modified S172; almost complete y ion series for the peptide (Carb stands for carbamidomethyl). These evidences indicate that S172 was O-GlcNac modified. **d**, **e** SW1990 cells with indicated WT or mutant Flag-PFKFB3 (**d**) or SW1990 and HPDE cells with indicated glucose concentrations (**e**) were cultured for 12 h under hypoxia or normoxia. The O-GlcNAc modified proteins modified by azide were labeled with biotin and isolated with streptavidin beads for immunoblotting analyses. In **a**, **b**, **d** and **e**, immunoblotting analyses were performed using the indicated antibodies.

Protein O-GlcNAcylation by OGT is important for cell proliferation, which may contribute to pancreatic tumorigenesis. To investigate how OGT is implicated in this process, O-GlcNAc-modified proteins from human pancreatic duct epithelial cancer cell lysates were labelled with non-natural azido sugar. Subsequent precipitation and immunoblotting showed that the PFKFB3, the hypoxia-induced regulator of glucose catabolism, is modified by O-GlcNAc, which was further enhanced by hypoxia in both SW1990 (Fig. 1a) and PANC-1 cells (Fig. S1a). To determine the mechanism, we stably expressed exogenous Flag-PFKFB3, the amount of which kept unchanged under hypoxia (Fig. 1b), in SW1990 cells. The followed analysis showed the O-GlcNAcylated Flag-PFKFB3, as well as the OGT protein level were also enhanced by hypoxia, both of which were negated by OGT shRNA (Fig. 1b), suggesting that the increased O-GlcNAcylation of PFKFB3 was not only due to the increased total amount of PFKFB3, but also the upregulated OGT activity during hypoxia. In line with previous report^4^, the global O-GlcNAcylation was also enhanced by hypoxia and further suppressed by OGT shRNA and glucose deprivation (Fig. S1b). Moreover, overexpressed OGT enhanced PFKFB3 O-GlcNAcylation in normal pancreatic duct epithelial (HPDE) cells (Fig. S1c, left), without affecting the PFKFB3 enzymatic activity (Fig. S1c, right).

To determine the PFKFB3 O-GlcNAcylation site, mass spectrometry^20^ analysis of Flag-PFKFB3 from SW1990 cells was performed. Several sites (Ser171, Ser172, Ser486, and Ser491) of PFKFB3 have been matched to O-GlcNac modification by software, and the highly credible modification at Ser172 was suggested by further analysis (Fig. 1c). In addition, mutagenesis studies showed the Ser171A/Ser172A largely abolished PFKFB3 O-GlcNAcylation under hypoxia, but not Ser486A/Ser491A (Fig. 1d), suggesting that PFKFB3 is mainly O-GlcNAcylated at Ser171/Ser172 under hypoxia. Furthermore, the limited glucose supply notably decreased the hypoxia-induced PFKFB3 O-GlcNAcylation in both cancer (SW1990) and normal (HPDE) cells, but still remained at a comparable level in cancer cells due to the aberrant OGT activity (Fig. 1e), which is in line with the global cellular O-GlcNAcylation according to our study (Fig. S1b) and previous report^3,4,21,22^.

### O-GlcNAcylation of PFKFB3 maintain cell cycle progression under hypoxia

**Fig. 2:**
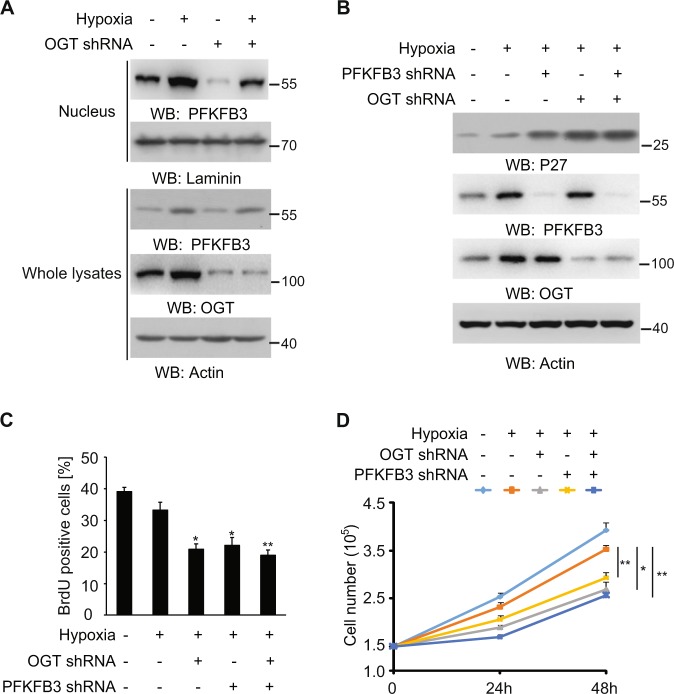
O-GlcNAcylation of PFKFB3 maintain cell cycle progression under hypoxia. **a**–**d** SW1990 cells with indicated shRNA expression were cultured for 12 h (**a**, **b**), 24 h (**c**), or indicated times (**d**) under hypoxia or normoxia; whole cellular (**a**, **b**) or nucleus extracts (**a**) were analyzed by immunoblotting; cellular proliferation rate was examined by BrdU incorporation assay (**c**) or cell number accounting (**d**). In **a**, **b**, immunoblotting analyses were performed using the indicated antibodies. In **c**, **d**, the values are presented as mean ± s.e.m. (*n* = 3 independent experiments), *represents *p* < 0.05 and **represents *p* < 0.01 (Student’s *t*-test) between the indicated groups.

To understand the biological role of the PFKFB3 O-GlcNAcylation, which exerts no effect on the enzymatic activity (Fig. 1c), we inhibited the O-GlcNAcylation with OGT shRNA. Under hypoxia, limited OGT activity showed no effect on the total PFKFB3 induction, but compromise its nuclear accumulation in both SW1990 (Fig. 2a) and PANC-1 cells (Fig. S2a). P27, which is upregulated by HIF-1α-dependent or independent pathways in different cell types under hypoxia, was generally reported as the essential mediator for the hypoxia-induced cell cycle arrest^23–26^. Several studies showed that the nuclear targeted PFKFB3 promotes the cell cycle progression mainly through degrading the P27 protein^18,19^. Here, in both SW1990 (Fig. 2b) and PANC-1 cells (Fig. S2b), the P27 protein was analyzed and slightly induced by hypoxia, but dramatically augmented by knocking down either PFKFB3 or OGT, with no additional effect when both were simultaneously depleted. Accordingly, BrdU incorporation (Fig. 2c) and cell accounting (Fig. 2d) analysis in tumor cells indicated that hypoxia slightly suppressed the proportion of divided cells, which was aggravated by PFKFB3 or OGT depletion, but without an additional effect when both were depleted. These results suggest that the O-GlcNAcylation of PFKFB3 is necessary to maintain cancer cell division under hypoxia via modulation of PFKFB3-P27 signaling pathway.

### OGT knockdown induces PFKFB3-G3BP2 interaction under hypoxia

**Fig. 3:**
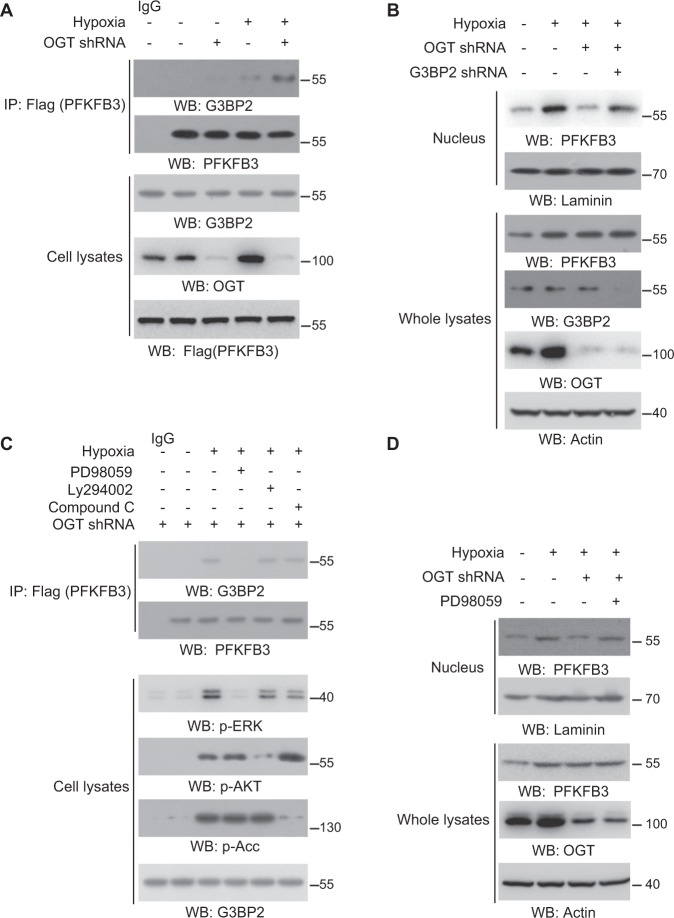
Limited O-GlcNAcylation induces PFKFB3-G3BP2 interaction under hypoxia. **a** SW1990 cells with Flag-PFKFB3 and indicated shRNA expression were cultured for 12 h under hypoxia or normoxia; whole cellular extracts subjected to immunoprecipitation with an anti-Flag antibody were analyzed by immunoblotting analyses. **b** SW1990 cells with indicated shRNA expression were cultured for 12 h under hypoxia or normoxia; whole cellular or nucleus extracts were analyzed by immunoblotting. **c** SW1990 cells expressing Flag-PFKFB3 and indicated shRNA were pretreated with PD98059 (10 μM), Ly294002 (10 μM) and Compound C (10 μM) for 1 h, before being cultured for 8 h under hypoxia or normoxia; whole cellular extracts subjected to immunoprecipitation with an anti-Flag antibody were analyzed by immunoblotting analyses. **d** SW1990 cells expressing indicated shRNA were pretreated with PD98059 (10 μM) for 1 h, before being cultured for 8 h under hypoxia or normoxia; whole cellular or nucleus extracts were analyzed by immunoblotting. In **a**–**d**, immunoblotting analyses were performed using the indicated antibodies.

To explore the underlying mechanism for the cytosolic retention of PFKFB3 in cells with limited OGT activity under hypoxia, we try to analyze the proteins which could interact with PFKFB3. Checking the previous MS analysis (Fig. 1d), we noticed the Flag-PFKFB3 precipitates contained the stress granule protein G3BP2, which was reported to function as cytoplasmic binding partner and prevent the nuclear translocation of multiple proteins^27–29^. The immunoprecipitation were performed to confirm that hypoxia slightly promoted the PFKFB3-G3BP2 complex formation, which could be dramatically enhanced by the OGT shRNA (Fig. 3a and Fig. S3). In consistent, OGT shRNA prevent the nuclear accumulation of PFKFB3 induced by hypoxia, which could be reversed by further knockdown of G3BP2 (Fig. 3b). These results indicate that O-GlcNAcylation maintains PFKFB3 nuclear localization through preventing PFKFB3-G3BP2 interaction.

To determine the mechanism for PFKFB3-G3BP2 interaction induced by hypoxia, we inhibited several pathways, which could be activated by hypoxia, with different inhibitors including AMPK inhibitor Compound C, ERK inhibitor PD98059 and AKT inhibitor Ly294002. We found hypoxia-induced PFKFB3 and G3BP2 interaction was effectively blocked by PD98059 (Fig. 3c). In line with this, ERK inhibition by PD98059 significantly reverse the OGT deficiency arrested- nuclear accumulation of PFKFB3 (Fig. 3d). This suggested that, with OGT knockdown, hypoxia-induced PFKFB3-G3BP2 interaction and PFKFB3 cytosolic retention through ERK pathway.

### ERK-mediated PFKFB3 phosphorylation at the O-GlcNAcylation site

**Fig. 4:**
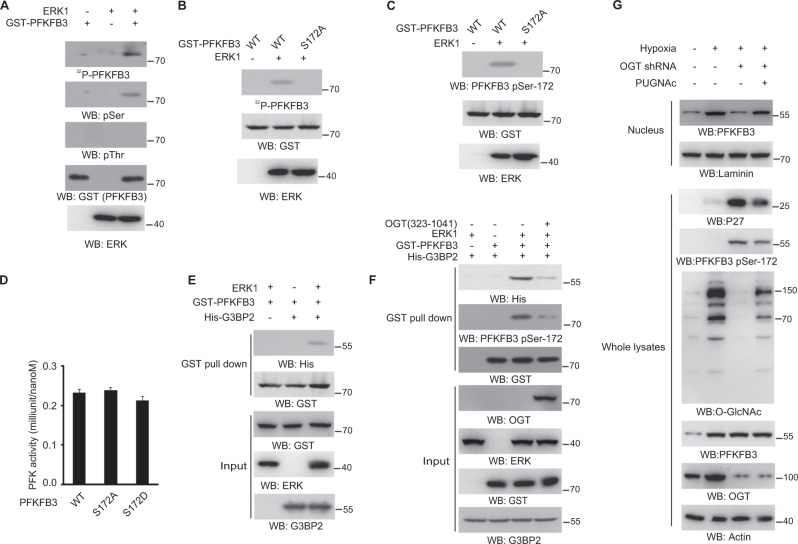
ERK-mediated PFKFB3 phosphorylation at the O-GlcNAcylation site. **a**–**c** In vitro phosphorylation analyses were performed by mixing the purified ERK1 with the indicated purified GST-PFKFB3 proteins or mutant proteins in the presence of [γ-^32^P]ATP (**a**, **b**). **d** The indicated purified PFKFB3 WT or mutant proteins were analysed by enzymatic activity assay. **e**, **f** The indicated purified GST–PFKFB3 protein, or pre-incubated with purified OGT (**f**), was mixed with purified His–G3BP2 protein with or without purified ERK1. GST pulldown analyses were performed. **g** SW1990 cells with indicated shRNA expression were pretreated with or without PUGNAc (20 μM) for 24 h and cultured for 12 h under hypoxia or normoxia. In **a**–**c**, and **e**–**g**, immunoblotting analyses were performed using the indicated antibodies. In **d**, the values are presented as mean ± s.e.m. (*n* = 3 independent experiments).

To further investigate the mechanism for ERK regulating PFKFB3-G3BP2 interaction, we tested whether PFKFB3 is the substrate of ERK. Interestingly, the in vitro protein phosphorylation assay showed the PFKFB3 could be directly phosphorylated by ERK1, which was detected only with a pan phosphoserine antibody (Fig. 4a). Additionally, several potential ERK phosphorylation residues at PFKFB3 were revealed by Scansite analysis, however, only the pre-identified O-GlcNAcylation site Ser172 can be surface accessible by ERK1 (Fig. S4). The subsequent, in vitro kinase assay showed the S172A mutant abolished ERK1-mediated PFKFB3 phosphorylation, as demonstrated by autoradiography (Fig. 4b) and immunoblotting analysis with a specific PFKFB3-pS172 antibody (Fig. 4c). However, the S172A mutant showed a similar activity to WT PFKFB3 and phosphor-mimic PFKFB3 S172D (Fig. 4d). Meanwhile, GST pulldown analysis showed GST-PFKFB3 could directly bind with His-G3BP2 in the presence of ERK1 (Fig. 4e), indicating PFKFB3 phosphorylation by ERK is sufficient for PFKFB3-G3BP2 complex formation. In addition, the PFKFB3 pre-incubated with OGT could not be phosphorylated by ERK1 and interacted with G3BP2 (Fig. 4f), suggesting the mutually exclusive modification of Ser172 residue by ERK1 or OGT determines the binding affinity of PFKFB3 with G3BP2. Moreover, the enhancement of cellular O-GlcNAcylation by PUGNAc in cancer cells with OGT knockdown, will notably reduce PFKFB3-S172 phosphorylation, increase PFKFB3 nuclear localization and block hypoxia-induced p27 accumulation (Fig. 4g).

### PFKFB3-S172 phosphorylation inhibit cell proliferation under hypoxia

**Fig. 5:**
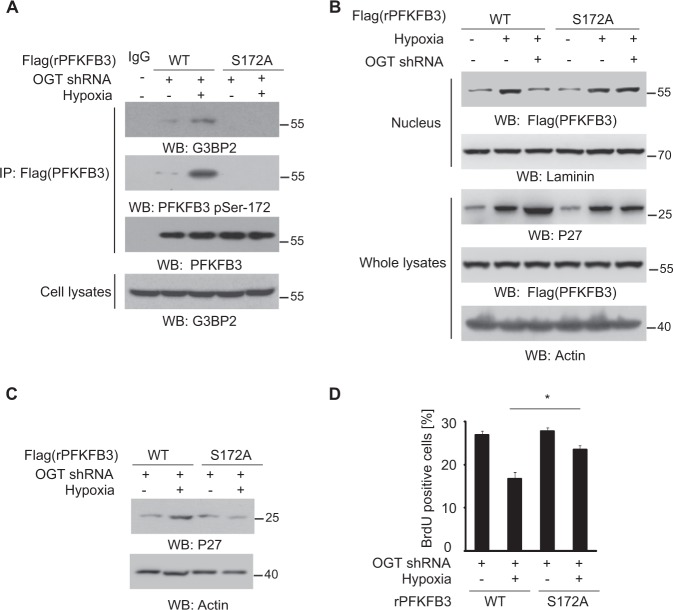
PFKFB3-S172 phosphorylation inhibits cell proliferation under hypoxia. **a**–**d** SW1990 cells were expressed with a vector for control shRNA or PFKFB3 shRNA and reconstituted with expression of rPFKFB3 WT or rPFKFB3 S172A. SW1990 cells expressing with indicated shRNA and indicated Flag-PFKFB3 were cultured for 12 h under hypoxia or normoxia; immunoprecipitation analyses (**a**) and immunoblotting (**a–c**) were performed. **d** SW1990 cells expressing with indicated shRNA and indicated Flag-PFKFB3 were cultured for 12 h under hypoxia or normoxia; cellular proliferation rate was examined by BrdU incorporation assay and FACS analyses. In **a**–**c**, immunoblotting analyses were performed using the indicated antibodies. In **d**, the values are presented as mean ± s.e.m. (*n* = 3 independent experiments), *represents *p* < 0.05 (Student’s t-test) between the indicated groups.

To confirm the role of PFKFB3-S172 phosphorylation in cancer cell proliferation, we depleted endogenous PFKFB3 in SW1990 cells, and reconstituted the expression of RNAi-resistant rPFKFB3 and rPFKFB3 S172A (Fig. S5). As results, rPFKFB3 showed remarkable PFKFB3-S172 phosphorylation and efficient interaction with G3BP2 under hypoxia, but not rPFKFB3 S172A (Fig. 5a). Cellular fraction analysis showed the PFKFB3 nuclear translocation under hypoxia was suppressed by OGT knockdown, in SW1990 cells with WT PFKFB3 but not PFKFB3 S172A (Fig. 5b). Consistently, hypoxia-induced P27 protein accumulation (Fig. 5c) and cell proliferation inhibition (Fig. 5d) were attenuated by PFKFB3 S172A, in SW1990 cells with OGT depletion. These results suggest inhibition of PFKFB3-S172 phosphorylation by OGT maintains cancer cell proliferation via degrading P27 under hypoxia. These results suggest O-GlcNAcylation is required for inhibition of PFKFB3-S172 phosphorylation under hypoxia, and further needed for cancer cell proliferation via degrading P27.

### Inhibition of PFKFB3-S172 phosphorylation by OGT is required for tumorigenesis

**Fig. 6:**
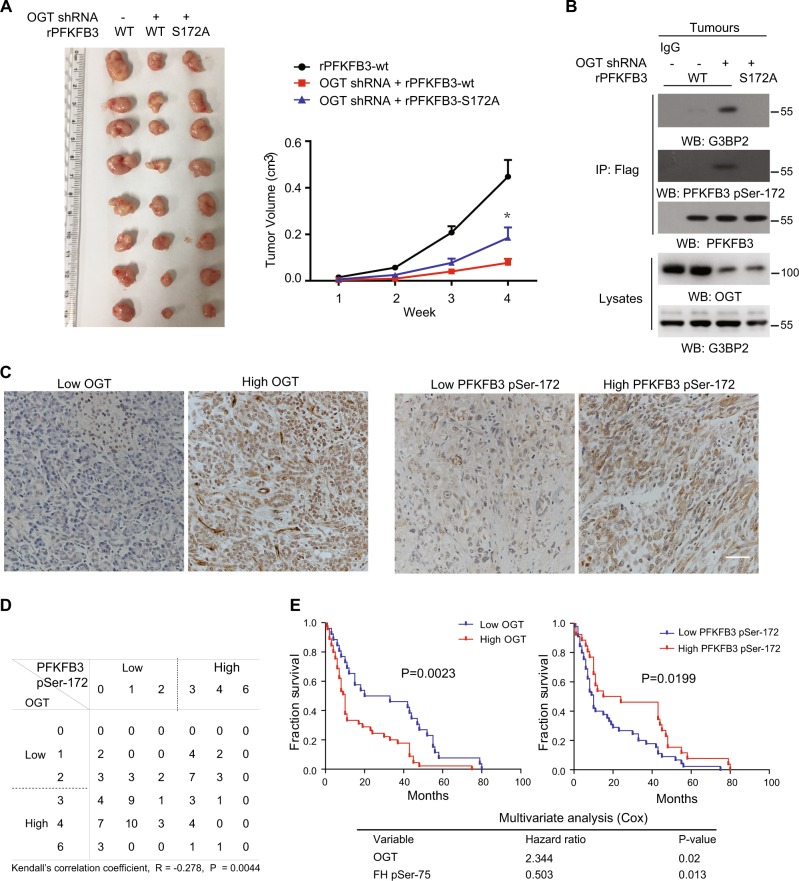
Inhibition of PFKFB3-S172 phosphorylation by OGT is required for tumorigenesis. **a** A total of 3 × 10^6^control or OGT depleted SW1990 cells with PFKFB3 depletion and reconstituted expression of the WT rPFKFB3 or rPFKFB3 S172A were subcutaneously injected into the athymic nude mice. Representative tumour xenografts were shown (left panel). Data represent the means ± s.e.m. (*n* = 8 mice per group, right panel). **b** Immunoprecipitated Flag-PFKFB3 from 8 pooled lysates of tumour tissues pool (as indicated in Fig. 6a) was subjected to immunoblotting analyses. **c** Immuno-histochemical staining was performed on 73 human pancreas tumour specimens. Representative images are shown. Scale bars: 50 μm. **d** Semi-quantitative scoring was performed (Kendall’s correlation test; R = −0.278, *P* = 0.0044.). **e** The survival times for 73 patients with low (0–2 staining scores, blue curve) versus high (3–6 staining scores, red curve) OGT (low, *n* = 26 patients; high, *n* = 47 patients) or with low (0–2 staining scores, red curve) versus high (3–6 staining scores, red curve) PFKFB3-S172 phosphorylation (low, *n* = 47 patients; high, *n* = 26 patients) (right) were compared. The Kaplan-Meier method and log-rank tests indicate the significance level of the association of OGT (*p* = 0.0023) with patient survival and the significance level of the association of PFKFB3-S172 phosphorylation (*p* = 0.0199) with patient survival. The table (lower) shows the cox-multivariate analysis after adjustment for patient sex and age, indicating the significance level of the association of OGT (*p* = 0.02, HR = 2.344) with patient survival duration and the significance level of the association of PFKFB3-S172 phosphorylation (*p* = 0.013, HR = 0.503) with patient survival duration.

To further investigate the functional role of PFKFB3 S172 phosphorylation on tumorigenesis, OGT-restrained SW1990 cells expressed with WT or S172A PFKFB3 were subcutaneously injected into athymic nude mice. SW1990 cells with limited OGT activity showed compromised tumorigenesis, which was reversed by PFKFB3 S172A (Fig. 6a). Meanwhile, immunoprecipitation analysis of tumor tissues, with WT PFKFB3 but not PFKFB3 S172A, showed the PFKFB3-S172 phosphorylation and PFKFB3-G3BP2 interaction were enhanced by OGT depletion (Fig. 6b). These data suggest hypoxia could be induced in vivo, which might be caused by the hypovascular state within the tumor mass.

High expression of OGT predicts poor prognosis in pancreatic cancer patients^30^. To determine the clinical relevance of our finding that OGT inhibits hypoxia-induced PFKFB3-S172 phosphorylation, we performed IHC to analyze OGT and PFKFB3 pSer172 levels in 73 human pancreatic tumor specimens with validation of the antibody (Fig. 6c and Fig. S6). Consistent with cell culture study, the quantified PFKFB3-S172 phosphorylation levels were inversely correlated with OGT in a significant value (Fig. 6d). In addition, the survival proportion of patients was calculated with respect to OGT or PFKFB3 pSer172 levels. Consistently^30^, patients with high OGT tumors (47 cases) had median survival duration of 10 months, which was significantly lower compared to 26.5 months for the patients with low OGT tumors. Oppositely, patients whose tumors had low PFKFB3 pSer172 (47 cases) had a median survival of 10 months compared to 19.5 months with high PFKFB3 pSer172 tumors (Fig. 6e). These results support the inhibitory role of hypoxia-induced PFKFB3 phosphorylation by OGT is clinical related to pancreatic tumorigenesis.

## Discussion

Cell division, which consumes vast amount of energy, must be coordinated with the glucose catabolism to meet the bioenergetic and biosynthetic demands. However, cancer cells, which frequently confront with limited oxygen and glucose due to the hypovascularity, have to keep cell devision for sustainable tumor growth. To deal with this dilemma, specific strategies are utilized to counter the microenvironment-induced energy pressures, such as potent glucose uptake, efficient glycolysis, alternative nutrient source from autophagy or macropinocytosis, and so on^8,31^. In addition, the signaling pathways brought up by the nutrient stress, which normally impedes cell division, have to be bypassed for cancer development. The O-GlcNAcylation, which plays as the sensor of oxygen and glucose as well as the regulator of cell proliferation^3,4,12,32^, might be the destined mediator for these processes.

Cells under hypoxia will take multiple responses, including drastic changes in signaling pathway and gene expression mediated by HIFs, resulting in cell cycle arrest and energy conservation^23^. One of the HIFs-induced gene in this response is PFKFB3. PFKFB3 is an unique glycolic enzyme, which contains nuclear localization signal (NLS; aa461-482). Although only the cytosolic fraction promotes energy production, PFKFB3 has been reported to localized in the nuclei without any treatment^33^. More nuclear roles of PFKFB3 have been reported, such as maintaining cell cycle^18,19^ and DNA stability^34^. Previous reports suggest that nuclear accumulation of Fru-2,6-BP caused by nuclear PFKFB3, will activate Cdk1 to phosphorylate the Thr187 site of P27 and cause it degradation, which couples the activation of glucose metabolism with cell proliferation^18,19^. Some strategies has been chose for cells to retain PFKFB3 in cytosol in stress conditions to fulfill cellular energy demands^33^. However, high O-GlcNAcylation, which usually means sufficient cellular energy source, might prefer to compromise such strategy to highlight the nuclear function of PFKFB3.

In our study, we firstly demonstrated that the PFKFB3 could be *O*-GlcNAcylated by OGT and phosphorylated by ERK at the same Ser172 residue, which are mutually exclusive and both induced by hypoxia; *O*-GlcNAcylated PFKFB3 freely shuttle to nucleus, in contrast, ERK-phosphorylated PFKFB3 is retained in cytosol by binding with G3BP2. The balance between these two modifications determines the distribution of PFKFB3, and presumably, coordinates the rate of cell devision with energy production. The inhibited O-GlcNAcylation keeps more retention of hypoxia-induced cytosolic PFKFB3; inversely, elevated O-GlcNAcylation is required to maintains more hypoxia-induced nuclear PFKFB3 and higher rate of cell division.

These results provide a delicate mechanism for O-GlcNAcylation at a threshold level to overcome signals responses to environmental stress, and also uncover a crucial role of PFKFB3 linking its metabolic function to the cell proliferation, which potentially provides a molecular basis for strategy against tumors with aberrant OGT activity and PFKFB3.

## Materials and methods

### Cell culture

HPDE cells were maintained in Dulbecco’s modified Eagle’s medium (DMEM) supplemented with 10% fetal bovine serum (FBS). SW1990 cells were maintained in RPMI 1640 medium supplemented with 10% FBS. All the cell lines were from ATCC and routinely tested for mycoplasma contamination.

Cell lines with OGT knockdown were generated as described previously^22^. The knockdown efficiency was examined by western blotting analysis for each picked cell strain pools.

### Hypoxic and glucose treatment

Hypoxia experiments were performed in a sealed hypoxia chamber (Proox Model 110, BioSpherix, Ltd.) filled with 1% O2, 5% CO2, and 94% N2 at 37 °C and 60% cell confluency for the indicated periods of time. For glucose treatment experiments, the cells were cultured in low glucose DMEM media (2 mM glucose) or high glucose DMEM media (25 mM glucose) for the indicated periods of time.

### DNA constructs and mutagenesis

The DNA constructs and mutagenesis were described as previously^22^. pGIPZ human OGT shRNA was generated with the oligonucleotides 5’-TTTATCAGGATTGTGCATG-3’ and 5’-AAATTGATATAAGCATCCA -3’. pGIPZ human PFKFB3 shRNA was generated with the oligonucleotide 5’-TTGAGAGATGTCAAAAGCT-3’ and 5’-AGGGACTTGTCGCTGATCA-3’. pGIPZ human G3BP2 shRNAs were generated with the oligonucleotides 5’-AGAAAGAAAGTTTATGCAA-3’ and 5’-AGAGGAGATATGGAACAGA-3’. The pGIPZ controls were generated with control oligonucleotide 5’-GCTTCTAACACCGGAGGTCTT-3’ or 5’-GCCCGAAAGGGTTCCAGCTTA-3’.

### Materials

Antibodies that recognize OGT (ab96718, 1:1000), G3BP2 (ab86135, 1:1000), Thiophosphate ester (ab92570, 1:1000) and Phosphoserine (ab9332, 1:1000) were purchased from Abcam. Antibodies that recognize Flag (#35535, 1:1000), Myc (#T504, 1:1000), His (#T505, 1:1000), GST (#T509, 1:1000) were obtained from Signalway Antibody. Antibodies against Flag (F3165, 1:1000), GST (G1160, 1:1000), and His (SAB4301134, 1:1000) were purchased from Sigma. Antibodies against PFKFB3 (D7H4Q, #13123, 1:1000), β-actin (#4970, 1:2000), p-Acc (#11818, 1:1000), O-GlcNAc (CTD110.6, #9875, 1:1000) were purchased from Cell Signaling Technology. Phospho-Erk (D155117, 1:1000) was purchased from Sangon Biotech. P27 (AP027, 1:1000) was purchased from Beyotime. FITC labeled antibody against BrdU (11-5071) were purchased from eBioscience. VeriBlot for IP Detection Reagent (HRP) (ab131366, 1:500-1:1000), the secondary antibody for immunoblotting with IP samples was purchased from Abcam; all other secondary antibodies (1:1000) were purchased from Beyotime.

Rabbit polyclonal PFKFB3 pSer172 antibody was made by Signalway Antibody. A peptide containing PFKFB3 pSer172 was injected into rabbits. The rabbit serum was collected and purified using an affinity column with non-phosphorylated PFKFB3 Ser172 peptide to exclude the antibodies for non-phosphorylated PFKFB3, followed by an affinity column with phosphorylated PFKFB3 Ser172 peptide to bind to and purify the PFKFB3 pSer172 antibody. The PFKFB3 pSer172 antibody was then eluted and concentrated.

PD98059, Ly294002, SB203580 were purchased from Cell Signaling Technology. Anti-FLAG^®^ M2 Beads, PUGNAc, Compound C, BrdU, EDTA-free Protease Inhibitor Cocktail, and PhosSTOP were purchased from Sigma. OGT and ERK1 were ordered from R&D. Hygromycin and puromycin were purchased from EMD Biosciences. Other materials were described previously^22^.

### Immunoprecipitation (IP) and immunoblotting analysis

Proteins were extracted from cultured cells using a modified buffer (50 mM Tris-HCl (pH 7.5), 1% Triton X-100, 150 mM NaCl, 1 mM DTT, 0.5 mM EDTA, and protease inhibitor cocktail or phosphatase inhibitor cocktail), followed by immunoprecipitation and immunoblotting with the corresponding antibodies. 3×Flag peptides were used to elute the precipitates from Anti-FLAG^®^ M2 Beads. For immunoblotting, cell lysate was prepared with cold RIPA lysis buffer. Nuclear extract was prepared by NEPERTM Nuclear and Cytoplasmic Extraction Reagents (Thermo) according to the manufacturer’s instructions. The protein concentration was determined using Bradford assay. Proteins from cell lysates or nuclear extracts were separated by SDS-PAGE, transferred onto PVDF membrane (Millipore) and probed with indicated antibodies.

### Mass spectrometry analysis

PFKFB3 modification and associated proteins were analysed by LC-MS/MS as described previously^22^. Briefly, Flag-PFKFB3 proteins from the immune-precipitation were acetone-precipitated in vitro at −20 °C overnight and resuspended in 50 mM ammonium bicarbonate buffer containing Rapigest (Waters Corp). The sample was heated to 95 °C for 10 min and allowed to cool down before 100 ng of sequencing-grade modified trypsin (Promega) was added. The digestion proceeded overnight at 37 °C and was analysed by LC-MS/MS using an Orbitrap-Elite mass spectrometer (Thermo Fisher Scientific). Proteins were identified by comparing the fragment spectra against those in the SwissProt protein database using Mascot v.2.3 (Matrix Science) and Sequest v.1.20 via Proteome Discoverer v.1.3 (Thermo Fisher Scientific) software.

### Recombinant protein purification

WT and mutant GST-PFKFB3 and His-G3BP3 were expressed in bacteria and purified, as described previously^22^. Briefly, corresponding constructs were used to transform BL21/DE3 bacteria. The cultures were grown at 37 °C to an OD600 of ∼ 0.6 before inducing with IPTG for 3 h. Cell pellets were collected and lysed by sonication. For GST-tagged proteins, cleared lysates were bound to glutathione-agarose. For His-tagged proteins, cleared lysates were bound to Talon metal affinity resin. Eluates were concentrated using Ultrafree-15 centrifugal filters (Millipore).

### O-GlcNAcylation assay

Enzymatic labelling and biotinylation of O-GlcNAc residues of cellular proteins were performed as described previously^22^. Briefly, cells were lysed in lysis buffer (50 mM Tris-HCl pH 7.5, 1% Triton X-100, 0.1% SDS, 150 mM NaCl, 0.5 mM EDTA, 1 mM DTT, 10 µM PUGNAc, and protease inhibitor cocktail). The cell lysate was enzymatically labelled with an azido-containing nucleotide sugar analogue (UDP-GalNAc) using an engineered β(1,4)-galactosyl transferase according to the Click-iT O-GlcNAc enzymatic labelling system protocol (Invitrogen) and conjugated with an alkyne-biotin compound following the Click-iT protein analysis detection kit protocol (Invitrogen). Biotinylation enabled the capture of O-GlcNAc-modified proteins from the lysate using streptavidin resin. Subsequent immunoblotting with an antibody against PFKFB3 was performed, to detect the O-GlcNAc modified PFKFB3 protein level.

### ERK treatment

Purified WT and mutant GST-PFKFB3 were incubated with ERK1 (R&D) in kinase assay buffer supplemented with 0.2 mM AMP and cold ATP in the presence or absence of 0.2 mCi/ml hot ATP (ICN Biochemicals) for 20 min at 30 °C. After the reaction, ERK1 was removed by extensive washing with RIPA buffer and kinase assay buffer. The GST-PFKFB3 bound beads were recovered by centrifugation. For kinase phosphorylation analyses, the GST-PFKFB3 bound beads were subjected to SDS-PAGE, and then autoradiography after incubation with EN3HANCE (PerkinElmer). The GST-PFKFB3 bound beads after ERK1 treatment in the absence of hot ATP were further incubated with His-G3BP2 or OGT for protein interaction assay as indicated.

### Transfection

HPDE and SW1990 cells were transfected with various plasmids using lipofectamine 3000 (Invitrogen) according to the vendor’s instructions.

### Cell proliferation assay

After treatment, cells were incubated with 50 µM BrdU for 1 h. Cells were collected by tryptase and centrifugation and fixed in 70% ethanol at 4 °C for 1 h and subsequently incubated with 2 N HCl/0.5%Triton X-100 for 30 min, 0.1 M borate sodium for 2 min. After anti-BrdU-FITC (eBioscience) incubation and washing, BrdU incorporation rate were analysed by flow cytometry.

### Enzyme activity assay

Measurement of PFKFB3 activity was performed by Phosphofructokinase (PFK) Activity Colorimetric Assay Kit (Sigma). Briefly, purified WT or mutant proteins were added to the PFK Assay Buffer, supplemented with PFK Enzyme Mix, PFK Developer, ATP and PFK Substrate. The blank was setted without PFK Substrate. PFK activity is determined by a coupled enzyme assay, in which fructose-6-phosphate and ATP is converted to fructose-1,6-diphosphate and ADP by PFK. The ADP is converted by the enzyme mix to AMP and NADH. The resulting NADH reduces a colorless probe resulting in a colorimetric (450 nm) product proportional to the PFK activity present. One unit of PFK is the amount of enzyme that will generate 1.0 μmole of NADH per minute at pH 7.4 at 37 °C. PFK activity is reported as nmole/min/mL = milliunit/mL. A standard curve was conducted with diluted NADH according to the kit. Incubate the plate at 37 °C and measure the absorbance at 450 nm every 5 min. The final measurement would be the penultimate reading. The enzyme activity was calculated according to the equation provided by the kit.

### Immunohistochemistry

Formalin fixed paraffin embedded consecutive human pancreatic cancer tissue sections (3–5 μm) were deparaffinized and rehydrated. Antigen retrieval was performed by boiling tissue sections in 10 mM citrate buffer (pH 6.0) in a microwave oven for 5 min. The activity of endogenous peroxidase was blocked with 3% hydrogen peroxide in methanol for 10 min atroom temperature. After washing, non-specific binding sites were blocked by incubating the slides with 10%FBS/PBS for 30 min at room temperature. Sections were subsequently incubated with rabbit polyclonal anti-OGT (ab96718, 1:100), anti-PFKFB3 pSer172 (Signalway, 1:50) at 4 °C overnight. After incubation with the primary antibodies, the sections were washed and incubated with secondary antibodies and DAB staining reagent with GTVision^TM^Detection System/Mo&Rb Kit according to manufacturer’s instructions. After counter stain with hematoxylin and dehydration, the sections were mounted and imaged using the Nikon DS-Ri2 microscope. Immunoreactivity was semi-quantitatively evaluated according to intensity and area: the staining intensity of pancreatic cancer cells themselves was recorded as “no staining (0)”, “weak to moderate staining (1)” or “strong staining (2)”. The area of stained cancer cells was recorded as < 33% (1), 33–66% (2) or > 66% (3) of all cancer cells. These numbers were then multiplied resulting in a score of 0–6. Statistical analysis of the survival time for 59 patients with low (scores ˂3) vs high (scores ≥ 3) OGT or PFKFB3 pSer172 levels, was performed using the GraphPad Prism 5 Software. The Kaplan-Meier method and log-rank tests were used for survival analysis. Statistical significance was set at *p* < 0.05.

The study was approved by the Ethics Committee of the First Affiliated Hospital of Wenzhou Medical University and patient consent was obtained.

### Mice

All animal experiments conformed to the guidelines of the Animal Care and Use Committee of Tianjin Medical University. Mice were maintained in a temperature- and light-controlled environment with ad libitum access to water. No blinding was done. The mice were randomly put into separate/groups cages for experiments and received a standard chow diet. Six-week-old male nu/nu mice (eight per group, the group sizes of the animals chosen are based on the numbers we used for previous publications, which is most optimal to generate statistically significant results.) were injected with 3 × 10^6^ gene-modified SW1990 cells in a volume of 150 μl of PBS. Injections were made subcutaneously in the left and right flanks, respectively at day 0. Tumor volume was measured by using length (a) and width (b) and calculated using the equation: V = ab^2^/2 with vernier calipers weekly. The samples for immunoprecipitation and immunoblotting analyses were from lysates of tumor tissues pool.

### Statistics and reproducibility

Statistical testing was performed using the Student’s *t*-test (unpaired two-tailed, unequal variance). The Kaplan-Meier method, log-rank tests was used for survival analysis. The data show normal distribution. All experiments were repeated at least three times unless otherwise indicated. N numbers are indicated in the figure legends. A *P*-value of 0.05 was considered as a border line for statistical significance.

## Supplementary information

Supplementary figures-ONCSIS-19-0454RRR

## Data Availability

Figures S1–S6 are attached. The original mass spectrometry data for PFKFB3 precipitates has been have been deposited in iProX (http://www.iprox.org) with the accession ProteomeXchange ID: PXD016644.
