# Supplementary material for: O-GlcNAcylation of PFKFB3 is required for tumor cell proliferation under hypoxia
Source: Oncogenesis. 2020 Feb 14;9(2):21. doi: 10.1038/s41389-020-0208-1 (PMC7021673; doi:10.1038/s41389-020-0208-1)
Supplement: Supplementary file 1 — Supplementary figures-ONCSIS-19-0454RRR [file 41389_2020_208_MOESM1_ESM.pdf]

Fig. S1

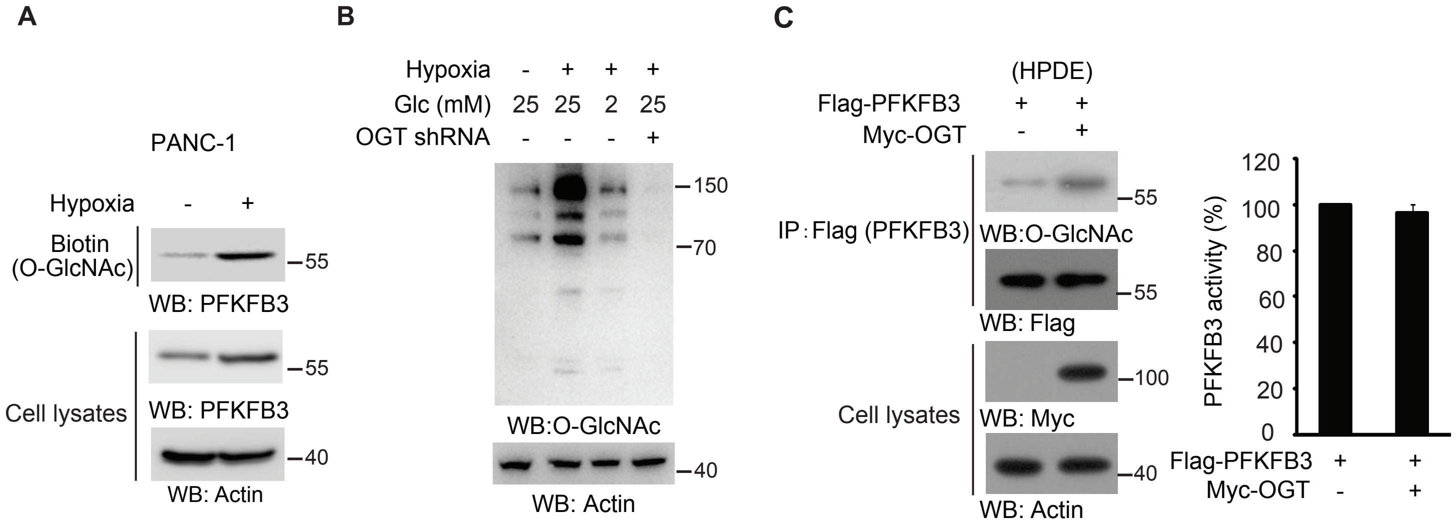

Fig. S1 PFKFB3 is modified by O-GlcNAc. (A) PANC-1 cells were cultured for 12 h under hypoxia or normal condition. The O-GlcNAc-modified proteins modified by azide were labeled with biotin and isolated with streptavidin beads for immunoblotting analyses with the indicated antibodies. (B) SW1990 cells with indicated glucose concentrations were cultured for 12 h under hypoxia or normoxia, whole cellular extracts were analyzed by immunoblotting.(C) HPDE cells expressing Flag-PFKFB3 was co-overexpressed with or without OGT. Whole cellular extracts subjected to immunoprecipitation with an anti-Flag antibody were analyzed by immunoblotting and enzymatic activity assay.

Fig. S2

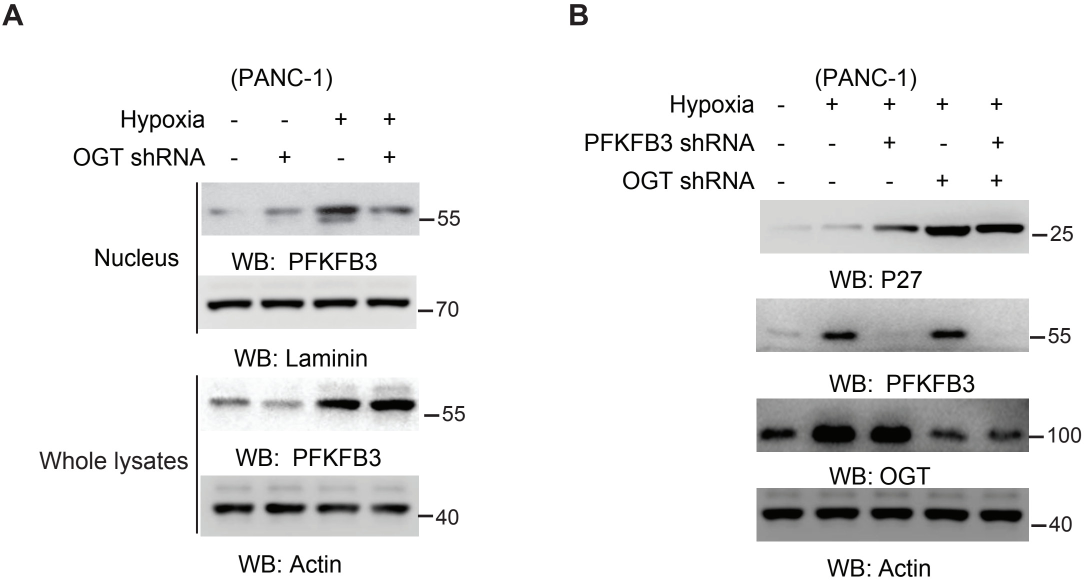

Fig. S2 O-GlcNAcylation of PFKFB3 maintain cell cycle under hypoxia. PANC-1 cells with indicated shRNA expression were cultured for 12 h in hypoxia or normal condition; whole cellular (A, B) or nucleus extracts (A) were analyzed by immunoblotting with the indicated antibodies.

Fig. S3

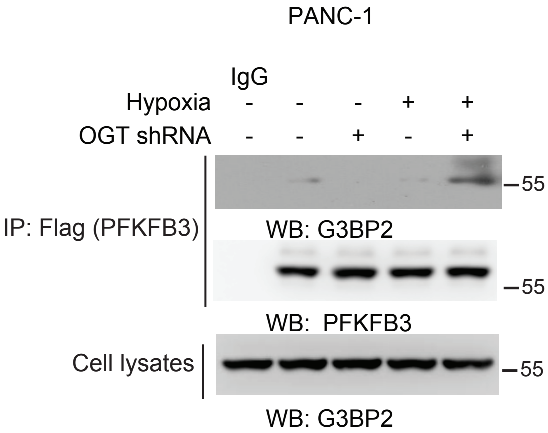

Fig. S3 Limited O-GlcNAcylation induces PFKFB3-G3BP2 interaction under hypoxia. PANC-1 cells with Flag-PFKFB3 and indicated shRNA expression were cultured for 12 h in hypoxia or normal condition; whole cellular extracts subjected to immunoprecipitation with an anti-Flag antibody were analyzed by immunoblotting analyses with the indicated antibodies.

Fig. S4

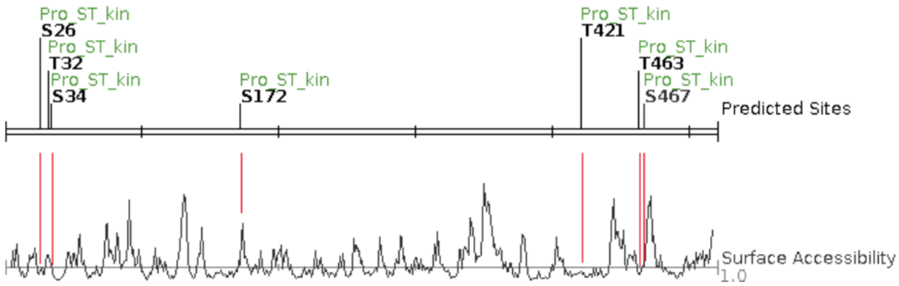

Fig. S4 ERK-mediated PFKFB3 phosphorylation at the O-GlcNAcylation site. ERK1 phosphorylation site at PFKFB3 were predicted by Scansite 4.0.

Fig. S5

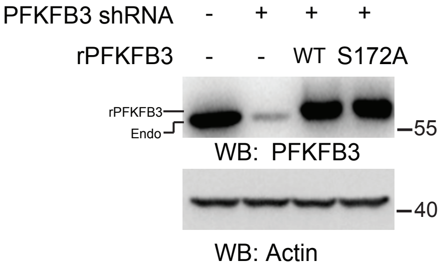

Fig. S5 PFKFB3-S172 phosphorylation inhibit cell proliferation under hypoxia. a SW1990 cells were expressed with a vector for control shRNA or PFKFB3 shRNA and reconstituted with expression of rPFKFB3 WT or rPFKFB3 S172A, immunoblotting analyses were performed using the indicated antibodies.

Fig. S6

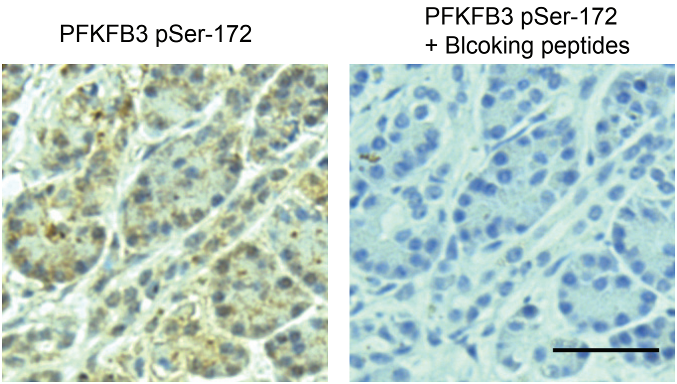

Fig. S6 Inhibition of PFKFB3 phosphorylation by OGT is required for tumorigenesis. The PFKFB3 S172 phosphorylation antibody specificities were validated using IHC analyses with specific blocking PFKFB3 S172 phosphorylation-peptides. Scale bars: 50 μm.
